# Study on leukapheresis of hyperleukocytic acute myeloid leukemia through in vitro centrifugation

**DOI:** 10.1186/s12885-024-12644-5

**Published:** 2024-07-24

**Authors:** Ruiyang Pan, Anjie Xu, Li Liu, Jinxian Wu, Xinqi Li, Guopeng Chen, Ruihang Li, Wanyue Yin, Dandan Liu, Xiaoyan Liu, Fuling Zhou

**Affiliations:** Department of Hematology, Zhongnan Hospital of Wuhan University, 169 Donghu Road, Wuhan, 430071 P. R. China

**Keywords:** Hyperleukocytic acute myeloid leukemia, Leukapheresis, In vitro centrifugation

## Abstract

Respiratory failure, intracranial hemorrhage and infection were more common in hyperleukocytic acute myeloid leukemia patients than in non-hyperleukocytic leukemia patients. Compared with non-apheresis treatment, the white blood cells decreased significantly and the infection rate decreased after apheresis treatment. However, the treatment time of leukapheresis in patients with hyperleukocytic leukemia is very long, while it is more damaging to cells. In this study, which conducted a retrospective analysis on patients with hyperleukocytic acute myeloid leukemia, the process of centrifugation of normal cells and patients’ cells by apheresis machine was simulated in vitro. Through selecting 5 healthy persons and 11 patients with hyperleukocytic acute myeloid leukemia, extracting their blood samples and performing in vitro centrifugation at different speeds or duration, we observed the changes of the numbers and morphology of peripheral blood cells in healthy people and patients, so as to explore the optimal centrifugation parameters during leukapheresis. The cells obtained by the optimal centrifugation parameters were cryopreserved and two groups of mice (10 mice in each group) were used to establish leukemia animal models. Through the research, it is found that when the centrifugal speed is below 6000 rpm, the damage to blood cells in healthy people and in patients with hyperleukocytic leukemia is not obvious. When the centrifugal speed is above 6000 rpm, the platelets will be damaged significantly. The cells obtained under the optimal centrifugation parameters can be successfully cryopreserved and used to establish leukemia animal models. This study is of great significance for improving the efficiency and reducing the side effects of leukapheresis, and is helpful to improve the treatment of white blood cells reduction.

**Supplementary Information:**

The online version contains supplementary material available at 10.1186/s12885-024-12644-5.

## Introduction

Acute myeloid leukemia(AML) is a common hematopoietic malignant tumor with high heterogeneity and biological complexity. Of the tens of thousands of new cases in the United States each year, nearly one-third of leukemia diagnoses are acute myeloid leukemia, and the incidence increases with age [1–3]. Hyperleukocytic acute myeloid leukemia is a high-risk type of AML, which is characterized by an abnormally high number of peripheral white blood cells, exceeding 100 × 10^9^/L [4, 5]. Hyperleukocytic acute myeloid leukemia accounts for 5% ~ 20% of adult AML [6], among which M2 and M5 are the most common types [7, 8]. If not treated actively, the 1-week mortality can reach 40% [8–10]. Severe hyperleukocytosis, which causes leukostasis, is a medical emergency with a risk of organ damage and is a poor prognostic factor for early death in patients with hyperleukocytosis [4, 11]. In clinical practice, leukostasis syndrome most commonly affects the lungs, central nervous system and kidneys, and is associated with pulmonary congestion infection, intracranial hemorrhage or infarction, melanoma, hematuria and other complications, which makes patients progress quickly, and increases the risk of death [9, 12]. However, leukostasis is a poorly understood and life-threatening complication of AML. Therefore, it is necessary to rapidly reduce white blood cells during the treatment. In addition, studies have confirmed that hyperleukocytosis is a poor prognostic factor for early death in hyperleukocytic leukemia patients, and the overall survival of patients with hyperleukocytic acute myeloid leukemia is low [8, 13]. These studies also suggest that patients with hyperleukocytosis who are suitable for chemotherapy may benefit from leukapheresis to prevent complications such as leukostasis that occur before AML is diagnosed and chemotherapy is started.

Leukapheresis is a type of physical therapy which reduces the number of white blood cells and blood viscosity in a patient’s blood [14], that is still the main treatment for patients with hyperleukocytic acute myeloid leukemia [15].

At present, there are other white blood cell reduction techniques in clinical practice, such as drug (using hydroxyurea) or intensive chemotherapy, which not only kills excess white blood cells, but also has a long treatment course [16]. In contrast, leukapheresis can quickly remove a large number of white blood cells, which effectively reduces the probability of leukostasis causing microthrombus in patients [17]. In addition, leukapheresis ensures the stability of blood circulation, while hydroxyurea or intensive chemotherapy is associated with multiple complications such as TLS, hyperkalemia, and acute renal failure [18].

However, leukapheresis therapy does not achieve the ideal effect at present [19], the reasons may be as follows: First, the time of leukapheresis is too long, which may delay the optimal treatment time of patients [20]. Second, the intervention of leukapheresis may be too late to reverse the cascade of events that have already begun due to hyperleukocytosis. Third, leukapheresis may damage blood cells, and ruptured cells releases a large number of harmful substances, which could aggravate the disease. Therefore, we should study the procedures of leukapheresis in more detail to improve the purity of blood cells in a short time.

Our study discusses whether it is possible to improve the separation efficiency by increasing the centrifugal speed while ensuring less cell damage, and to shorten the time of apheresis treatment and reduce white blood cells more quickly. Therefore, how to reduce the damage to cells and increase the centrifugal speed within a safe parameter range has become a major problem we have encountered.

Therefore, our study is dedicated to solving the current problems of apheresis, finding the optimal centrifugation parameters to improve the efficiency of apheresis treatment, gaining time for further treatment, and further improving the survival rate of patients with hyperleukocytic acute myeloid leukemia. In this study, we explored the effect of centrifugal forces on the numbers and morphology of peripheral blood cells in healthy people and patients with hyperleukocytic acute myeloid leukemia after in vitro centrifugation, hoping to find the optimal centrifugation parameters to improve the centrifugal speed without damaging normal cells, so as to achieve the purpose of improving the efficiency of apheresis.

## Materials and methods

### Patients

Five healthy persons donated blood. Inclusion criteria :1) Age: adult (≥ 18 years old); 2) Normal coagulation function; 3) No genetic history. Exclusion criteria :1) Drinking alcoholic beverages within 1 day before blood donation; 2) Take aspirin and other antiplatelet and anticoagulant drugs within 2 weeks before blood donation.

Eleven female patients with hyperleukocytic acute myeloid leukemia were selected, and peripheral blood of these patients was extracted with 10 tubes (2 ml each tube). There were not patient to patient variations at the same speeds (the same gender). It was extracted by the nurse and placed in purple, green and blue anticoagulant tubes. The donors for the study signed a written informed consent prior before having their blood drawn. The research program of blood donors has been approved by the local ethics committee, which is in line with China’s blood donation guidelines and in line with ethics.

### Equipment and reagents

The main equipment used in the experiment included centrifuge ST-8R (purchased from Thermo, USA), cell counter (purchased from Thermo, USA), flow cytometer (purchased from Beckman, Germany), etc. Reagents used included FBS, PBS, RPMI-1640 medium, and DMEM medium (all purchased from Gibco).

### Methods

Blood samples from each healthy person or patient were centrifuged at different centrifugal speeds, and then the centrifugal duration was changed at the same centrifugal speed. All centrifugation experiments were repeated for more than 3 times (the blood samples of multiple repeated experiments were from the same source), and there was no statistical difference in the results of multiple centrifugation after statistical analysis. Then, the average value of multiple centrifugation results was taken to ensure the consistency and repeatability of results.

### Whole blood cells were centrifuged at the same duration and at different centrifugal speeds

First of all, centrifuge at 0 rpm (non-centrifugation), 1500 rpm, 3000 rpm, 4500 rpm, 6000 rpm, 7500 rpm, 9000 rpm, 10,500 rpm, 12,000 rpm for ten minutes in centrifuges imported from the regular company (Thermo company). Then centrifuge at room temperature at 3000 rpm, 6000 rpm, 9000 rpm for 10 min, 20 min, 30 min, 40 min. The sample was kept consistent from the beginning of processing to the detection time, and the centrifuge temperature was 20℃.

### Processing centrifuged blood samples

The blood samples after treatment were divided into three parts. One part of each group was divided into three levels, which were red blood cells, white blood cells and platelets, and plasma. The second part was separated and added red blood cell lysate into the white blood cells and platelets to obtain relatively pure white blood cell and platelets after removing supernatant. The other part was mixed and tested for blood routine and other items.

### The cell numbers and morphology, biochemical examination and electron microscope morphology

A part of human whole blood was collected before and after centrifugation to detect blood routine, electrolyte, coagulation and other indexes and to observe red blood cell morphology. Cell apoptosis was detected in the cell suspension obtained by centrifugation. The platelet activation rate was measured by flow cytometry after treatment with different centrifugal parameters. The morphology of red blood cells (pre-diluted), white blood cells and platelets were observed under transmission electron microscope.

### Chromosome breakage experiment

Peripheral blood samples after centrifugation were added to 3 identical culture medium, and MMC was added to make the final concentrations of 0ng/ml, 50ng/ml and 100ng/ml, respectively. After a series of operations such as culturing and preparing, chromosome breakage was observed under microscope.

### Observation of cell microstructure

Homogeneous trace cells were recorded by taking photos, and the average cell sizes were measured. Through that, the changes of cell size were measured by calculating the average cell radius, and the changes in cell center point, brightness, image contrast and other changes were calculated to understand the changes of cell surface. In addition, the red blood cells, white blood cells and platelets after centrifugation were separated and labeled, and the cell morphology was recorded by microscopic single-cell imaging technology. The obtained cell images were processed and analyzed by computer.

### Cell cryopreservation was performed under the optimal centrifugation parameters

Peripheral blood samples of newly diagnosed patients with hyperleukocytic acute myeloid leukemia (M5) requiring cryopreservation were collected. The experimental group used the optimal centrifugation parameters previously explored, and the control group was centrifuged at 3000 rpm for 10 min. After centrifugation, the mononuclear cells were absorbed and mixed with the cleaning solution. The treated mononuclear cells were transferred to a sterile cryopreserved tube, and autologous plasma and DMSO (the ratio is 9:1) were added for cell cryopreservation. After 1 month, the frozen tubes were taken out and placed in 37℃ water bath for cell recovery. The supernatant was removed by centrifugation and the cells were stained with trypan blue. The cell survival rate was calculated three times to take the average value. After 6 months of frozen storage, the frozen tubes were taken out and placed in 37℃ water bath for cell recovery. After centrifugation, the uniformly obtained cell suspension was re-suspended with DMEN medium and cultured with CCK8 to detect the cell survival rate. Three times the volume of red cell lysate was added to the cell suspension obtained by centrifugation to detect the cell apoptosis.

### Human acute myeloid leukemia mouse model was established with the cells obtained under the optimal centrifugation parameters

Experimental cells were taken from the above frozen cells and divided into experimental and control groups according to whether or not centrifugation was performed with the optimal parameters. The experimental animals were immunodeficient B-NSG mice (qualification number: SCXK(Beijing)2022-0004) at 4–5 weeks of birth. One week after purchase, these NSG mice (female, 16–20 g, 5 weeks) were divided into two groups (10 mice in each group). They were given 2.0 Gy X-ray irradiation, and 2.0 × 10^7^ human acute myeloid leukemia cells (CD33CD117) were injected through the tail vein within 24 h to establish the human acute myeloid leukemia mouse model and observe them such as survival curves and body weight changes. The experiment lasted for 25 days. The mice were killed after the experiment, and the survival curves of each group were recorded. The mouse bone marrow was collected and the erythrocytes were lysed after the bone marrow was rinsed. Then the mononuclear cells in the bone marrow were collected, and flow cytometry was performed to detect the changes of the proportion of original cells in the bone marrow of mice in each group. The expression of CD117 in leukemia xenografts was detected by immunohistochemistry.

### Statistics

The significance of differences between two groups was determined using unpaired two-tailed Student t tests. All results in bar graphs are mean value ± SEM. Overall survival curves were plotted according to the Kaplan-Meier method with the log-rank test applied for comparisons. *P < 0.05, **P < 0.01, ***P < 0.001.

## Results

### The centrifugal speed affects the morphology of red blood cells, but does not affect the number of red blood cells

**Fig. 1:**
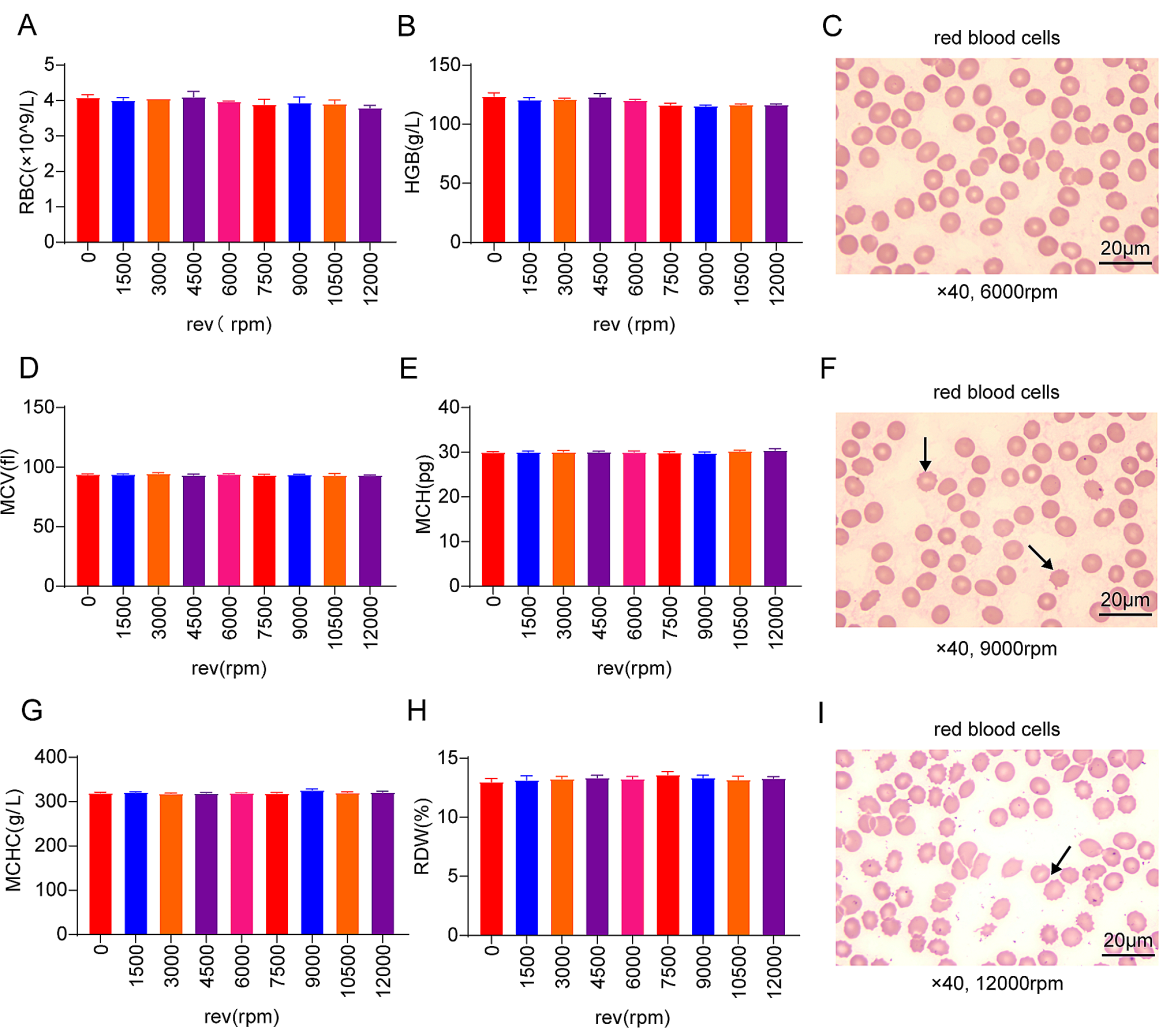
(**A**) The number of red blood cells at different centrifugal speeds (**B**) The content of hemoglobin at different centrifugal speeds (**C**) Morphology of red blood cells under 6000 rpm centrifugal force (**D**) mean corpuscular volume at different centrifugal speeds (**E**) mean corpuscular hemoglobin at different centrifugal speeds (**F**) Morphology of red blood cells under 9000 rpm centrifugal force (**G**) mean corpuscular hemoglobin concentration at different centrifugal speeds (**H**) Red blood cell distribution width at different centrifugal speeds (**I**) Morphology of red blood cells under 12,000 rpm centrifugal force

There was no difference in the number of RBC (Fig. 1A), HGB (Fig. 1B), MCV (Fig. 1D), MCH (Fig. 1E), MCHC (Fig. 1G) and RDW (Fig. 1H) under different centrifugal speeds (*P* > 0.05).

Red blood cells at 9000 and 12,000 rpm showed burr changes under microscope (Fig. 1F, I). The morphology of red blood cells at 6000 rpm was normal (Fig. 1C). Therefore, we know that the red blood cell morphology is normal at 6000 rpm and the cell membrane is intact, while the red blood cell morphology is wrinkled and spiny at 9000 rpm and 12,000 rpm. So we speculate that the centrifugal speed has some effect on the morphology of red blood cells, but does not change the number of red blood cells.

### The centrifugal speed and duration basically did not damage the white blood cells

**Fig. 2:**
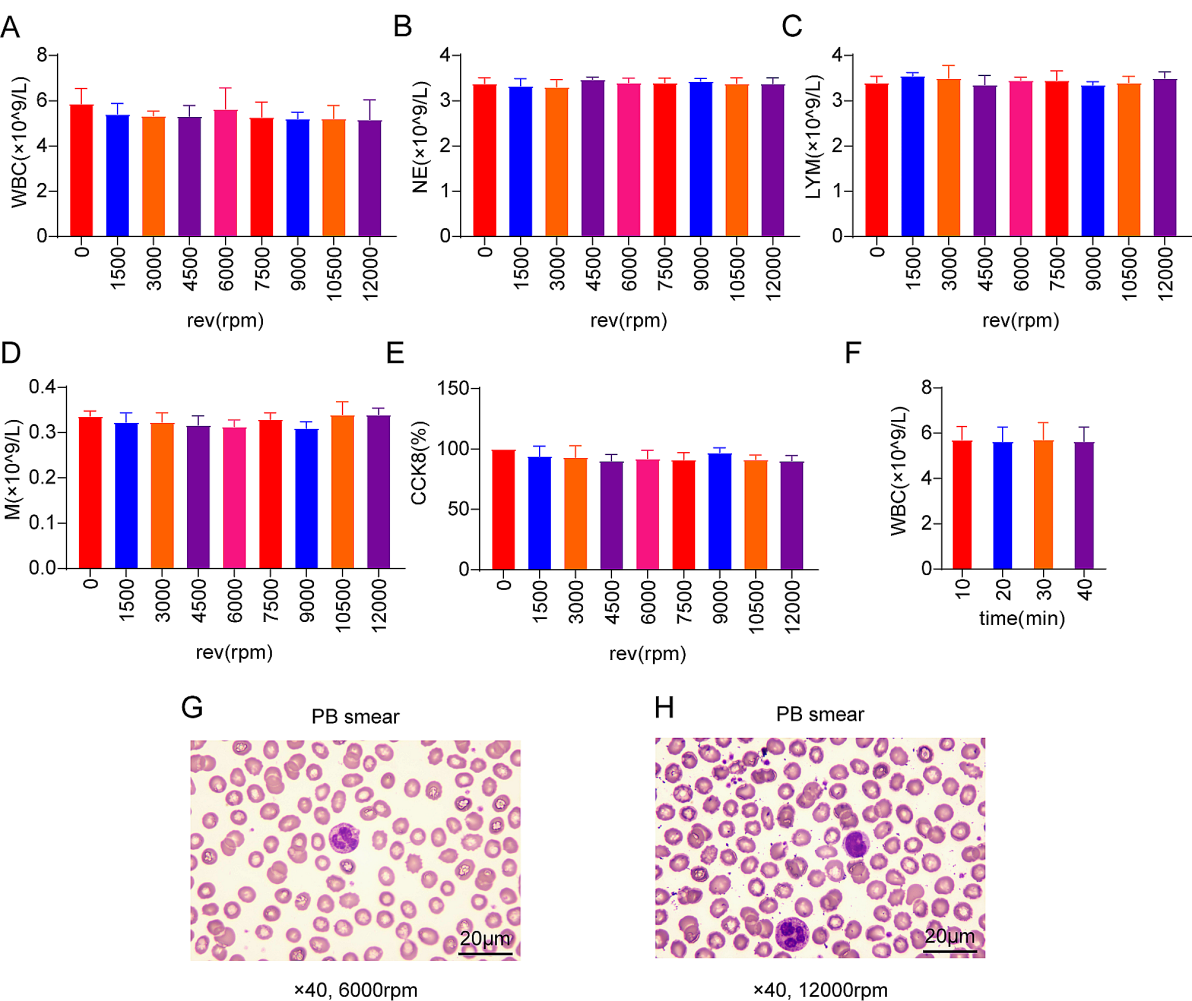
(**A**) The number of white blood cells at different centrifugal speeds (**B**) The number of neutrophils at different centrifugal speeds (**C**) The number of lymphocyte at different centrifugal speeds (**D**) The number of monocytes at different centrifugal speeds (**E**) Mononuclear cell activity at different centrifugal speeds (**F**) The number of white blood cells at different centrifugal duration (**G**) Peripheral blood smear under 6000 rpm centrifugal force (**H**) Peripheral blood smear under 12,000 rpm centrifugal force

There were no significant differences in the number of white blood cells (Fig. 2A), neutrophils (Fig. 2B), lymphocytes (Fig. 2C) and monocytes (Fig. 2D) under different centrifugal speeds (*P* > 0.05).

We can not observe the significant differences in the activity of peripheral blood mononuclear cells obtained at different centrifugal speeds, and the activity was about 90%. The activity of peripheral blood mononuclear cells obtained by centrifugation at 6000 rpm had little difference with that of centrifugation at 1500 rpm and 3000 rpm (Fig. 2E). There was no significant difference in the number of white blood cells at 10 min, 20 min, 30 min and 40 min (Fig. 2F). The morphology of white blood cells was normal under microscope (Fig. 2G, H). Therefore, we know that the centrifugal speed and duration have little effect on the damage of white blood cells.

### When the centrifugal speed was 6000 rpm, platelets could maintain low activation rate and normal morphology, but the activation rate increased with longer time

**Fig. 3:**
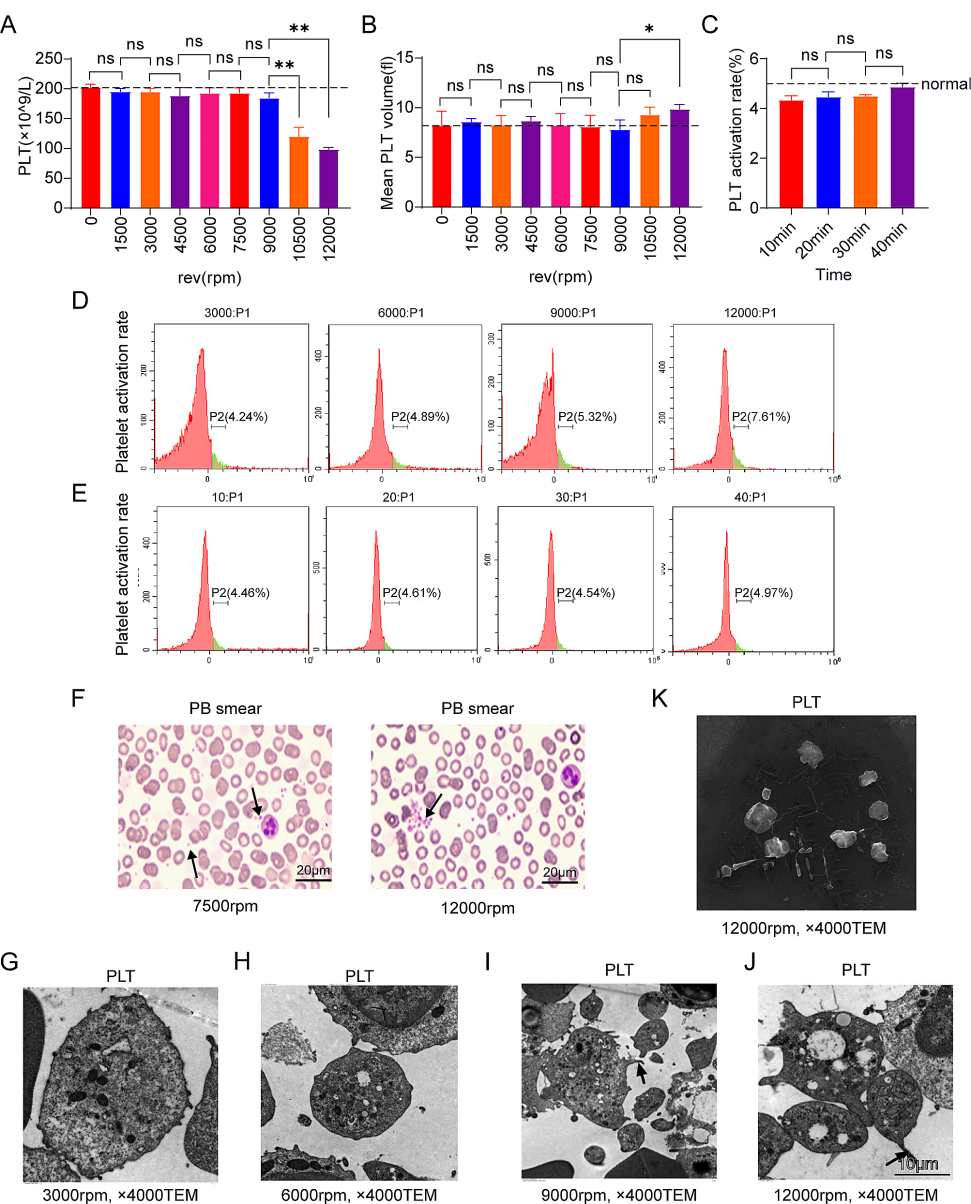
(**A**) The number of platelets at different centrifugal speeds (**B**) Average platelet volume at different centrifugal speeds (**C**) Platelet activation rate at different centrifugal duration (**D**) Platelet activation rate at different centrifugal speeds (**E**) Platelet activation rate at different centrifugation duration under 1500 rpm centrifugal force (**F**) Peripheral blood smears under centrifugal force of 7500 rpm and 12,000 rpm (**G** - **K**) Platelet morphology under electron microscopy under different centrifugal forces

The number of platelets decreased at 10,500 rpm (*P* < 0.05), while it also decreased at 7500 and 9000 rpm but there was no significant difference. At 1500, 3000, 4500, and 6000 rpm, platelets did not decrease significantly (Fig. 3A). The mean platelet volume increased at 12,000 rpm (*P* < 0.05), with significant differences (Fig. 3B). Platelet volume also increased at 10,500 rpm, but there was no significant difference. We observed by flow cytometry that the platelet activation rate was gradually increased. Platelet activation in the normal body cannot exceed 5%, so 9000 rpm and 12,000 rpm did not meet the requirements. At 6000 rpm, platelet activation did not exceed 5%, which was within the normal range (Fig. 3D). After centrifugation for 10, 20, 30, and 40 min at 1500 rpm, platelet activation rates gradually increased, but all were within the normal range (Fig. 3E). Figure 3C also shows that platelet activation did not exceed the normal range at 10, 20, 30, and 40 min centrifugation. Figure 3F shows platelet aggregation in the 7500 and 12,000 rpm groups in peripheral blood smears.

When the centrifugal speeds were 3000 rpm and 6000 rpm, the platelets had normal morphology under electron microscope, with no foot process, fewer intracellular Alpha particles and fewer mitochondrial vacuolation, and the platelets had elongated foot processes morphology at 7500 rpm and 9000 rpm. At 12,000 rpm, the number of Alpha particles increased, the platelet aggregation increased, and the intracellular vacuolation became larger and more numerous. Meanwhile, there were multiple ruptures of platelet membrane and mitochondrial vacuolation (Fig. 3G, H, I, J).

Observation under scanning electron microscopy showed that after centrifugation at 12,000 rpm the fibrin contained a large number of clustered white blood cells, and the fibrin matrix was interleaved in a grid pattern and wrapped white blood cells and platelets (Fig. 3K).

### Coagulation factors decreased gradually with the increase of the centrifugal speed

**Fig. 4:**
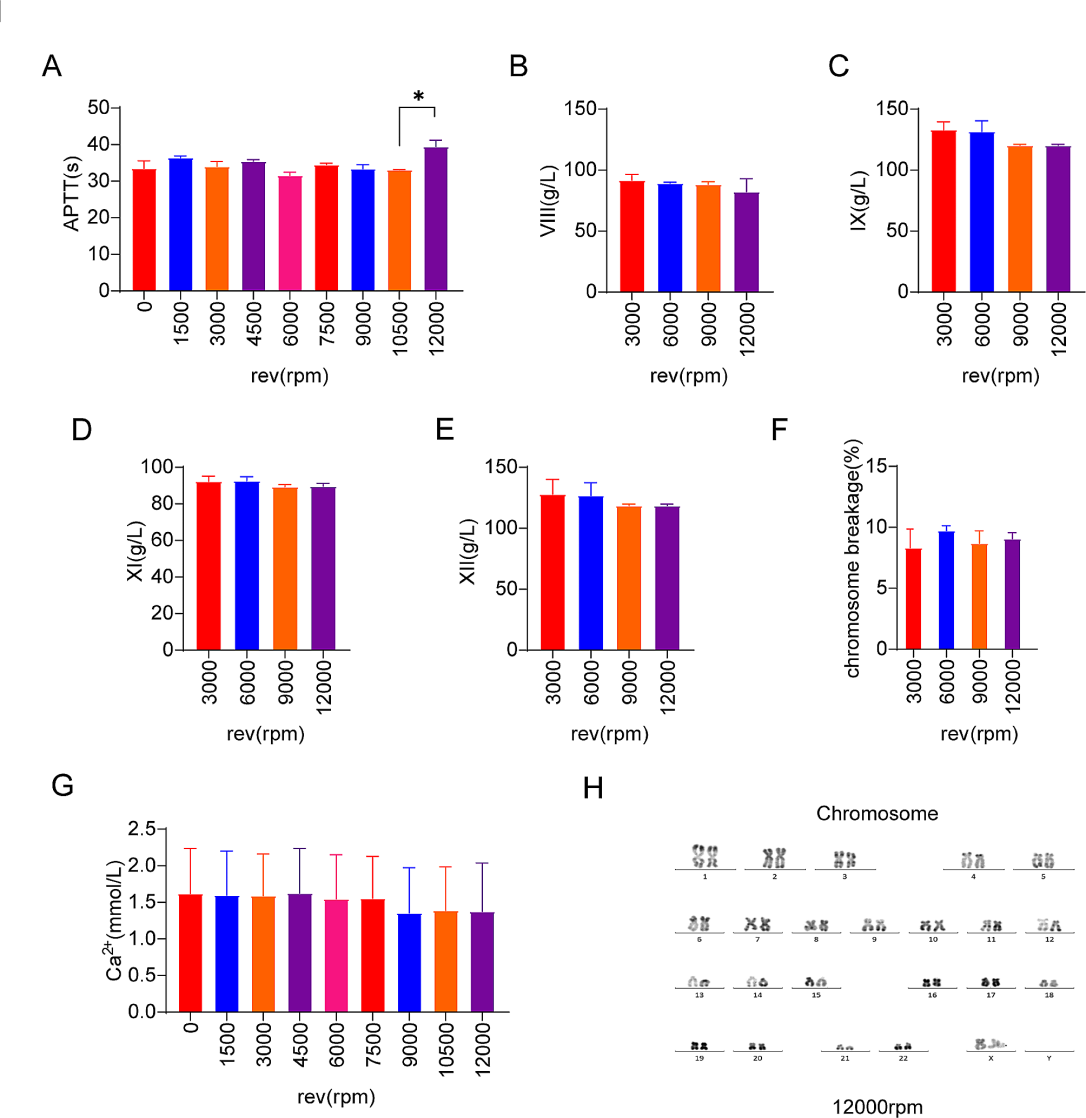
(**A**) APTT at different centrifugal speeds (**B**-**E**) Coagulation factor content at different centrifugal speeds (**F**) Chromosome breakage rate at different centrifugal speeds (**G**) Calcium ion content at different centrifugal speeds (**H**) Chromosome structure and morphology under 12,000 rpm centrifugal force

With the increase of the centrifugal speed, we can see a gradual decline in factors VIII, IX, XI and XII (Fig. 4B, C, D, E), and APTT lengthens at 12,000 rpm (*P* < 0.05) (Fig. 4A). Therefore, we speculate that endogenous coagulation pathways are activated at centrifugal speeds above 9000 rpm. Combined with Figs. 3 and 4, it is shown that different centrifugal speeds may change the structural changes within platelets, causing platelets to aggregate and activate, and affecting coagulation factors.

### The centrifugal speed did not affect the structure and morphology of chromosome

The incidence of chromosome breakage did not change much when the centrifugal speeds were 3000 rpm, 6000 rpm, 9000 rpm and 12,000 rpm, and it could be considered that different centrifugal speeds did not affect the structural and morphological of chromosomes in the cell nucleus (Fig. 4F, H,). What should be added is that calcium ions did not decrease significantly under different centrifugal speeds (Fig. 4G), which may be due to the influence of EDTA and sodium citrate in the blood collection tube, so the results may not be representative.

### The optimal centrifugation parameters were 6000 rpm, 10 min

**Fig. 5:**
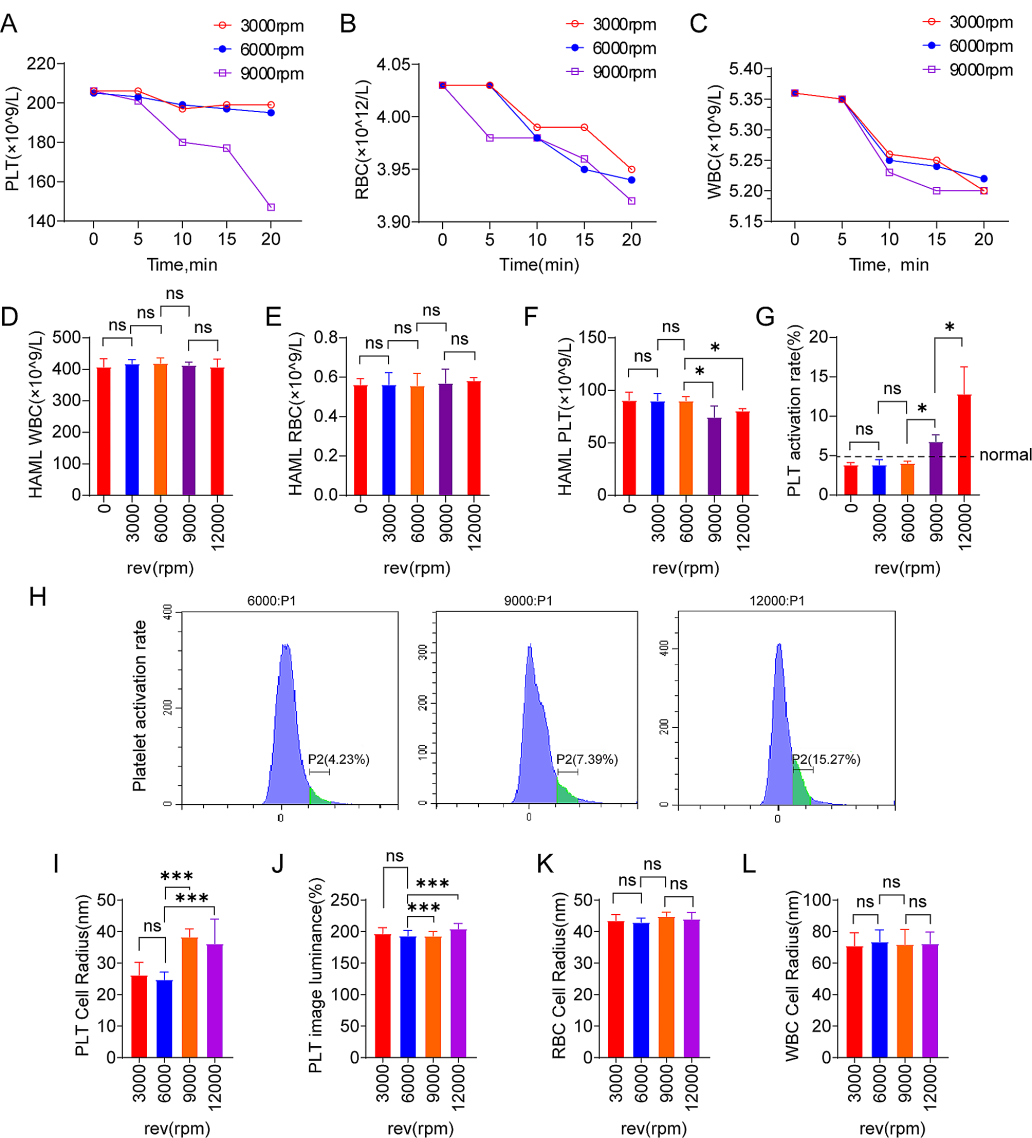
(**A**) The number of platelets at different centrifugal speeds (**B**) The number of red blood cells at different centrifugal speeds (**C**) The number of white blood cells at different centrifugal speeds (**D-F**) The number of white blood cells, red blood cells or platelets in peripheral blood of patients with hyperleukocytic acute myeloid leukemia patients (**G**, **H**) Platelet activation rate in peripheral blood of patients with hyperleukocytic acute myeloid leukemia patients (**I**) PLT cell radius at different centrifugal speeds (**J**) PLT image luminance at different centrifugal speeds (**K**) RBC cell radius at different centrifugal speeds (**L**) WBC cell radius at different centrifugal speeds

From our above experimental results, we can see that under different centrifugal speeds and duration, the number decrease and cell damage of red and white blood cells were little. For platelets, the decrease at 3000 and 6000 rpm was relatively little, and there was no significant difference between at 3000 rpm and 6000 rpm (Fig. 5A, B, C). However, when the centrifugal speed increased to 7500 rpm, the platelet activation rate exceeded 5%. The number of platelets decreased significantly at 10,500 rpm. From these results, we can see that the centrifugal speed of 6000 rpm had little damage to cells, and the platelet activation rate increased with the increase of the centrifugal duration. At this time, we found that the optimal centrifugation parameters were 6000 rpm,10 min.

### At 9000 rpm and above, the damage to platelets was great, but the effect on red blood cells and white blood cells was little

**Fig. 6:**
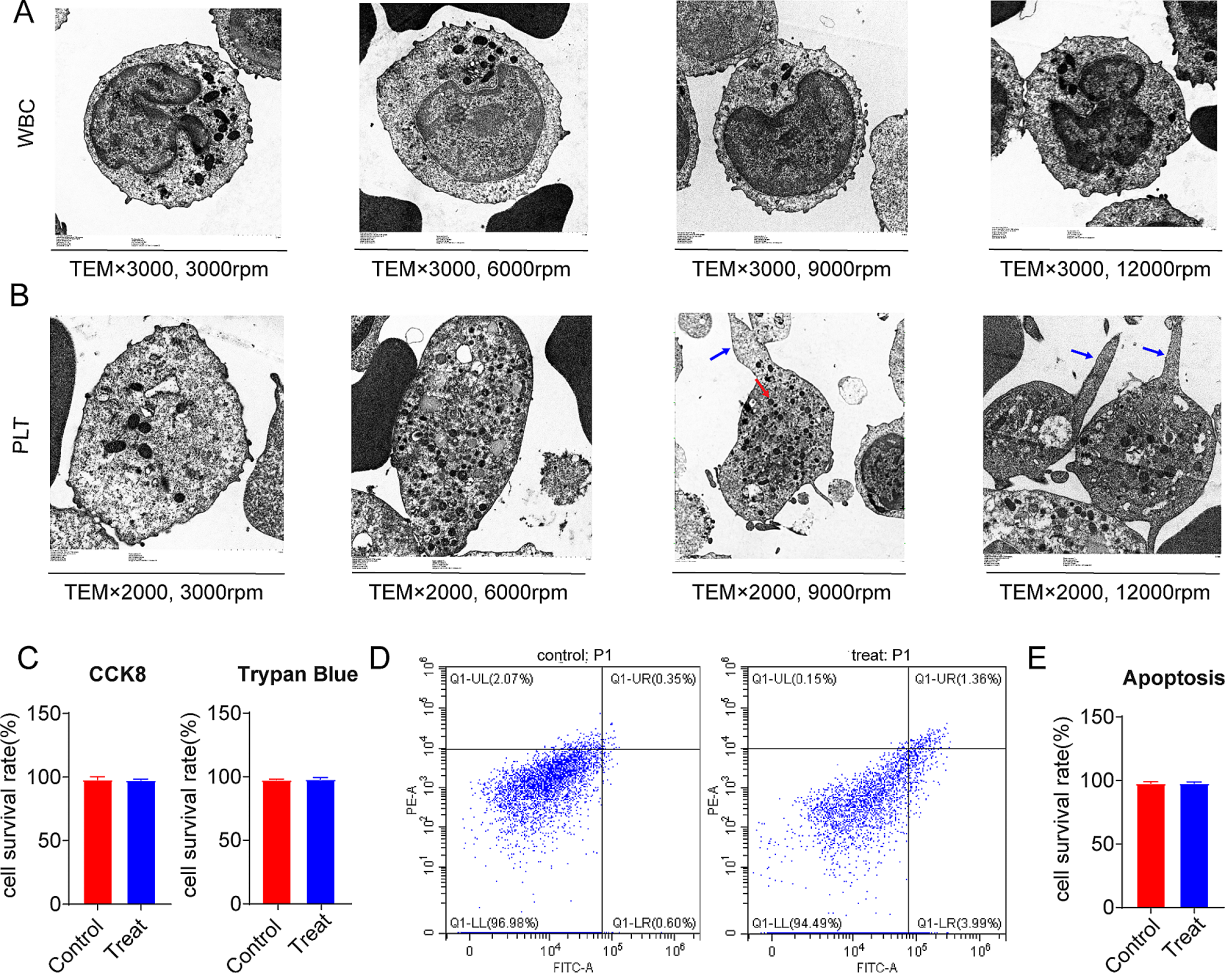
(**A**) Morphology of white blood cells under electron microscopy at different centrifugal speeds (**B**) Platelet morphology under electron microscopy at different centrifugal speeds (**C**) Cell survival rate of control and experimental groups after CCK8 and trypan blue treatment (**D**) Flow cytometry of cell apoptosis in the control and experimental groups (**E**) Cell apoptosis of control and experimental groups

Platelets also decreased significantly when the centrifugal speed was changed to 9000 rpm. However, different centrifugal speeds had little effect on red blood cells and white blood cells in hyperleukocytic acute myeloid leukemia patients. At 3000 and 6000 rpm, the platelet activation rate was in the normal range, while at 12,000 rpm platelets showed a large amount of activation and decreased in number (Fig. 5D, E, F, G, H). After the centrifugal speeds were 3000 rpm, 6000 rpm and 12,000 rpm, there was no statistical difference in the morphological changes of red blood cells and white blood cells in the peripheral blood of hyperleukocytic acute myeloid leukemia patients, such as mean cell radius. After centrifugated at 12,000 rpm, platelets clustered a lot, resulting in increased mean cell image brightness, larger mean image energy gradient, and larger mean cell radius (Fig. 5I, J, K, L). The morphology of white blood cells, such as neutrophils, lymphocytes and monocytes, did not change significantly under electron microscopy whether centrifugation was performed at a low centrifugal speed below 6000 rpm or at a high centrifugal speed such as 12,000 rpm (Fig. 6A). Under electron microscope, the number of Alpha particles in platelets was higher when the centrifugal speed was more than 6000 rpm. At 12,000 rpm, there were more platelet foot process and more platelet aggregation. This indicated that platelets were activated more thoroughly, which was more conducive to platelet aggregation, adhesion and other functions (Fig. 6B).

### Cell cryopreservation and establishment of leukemia mouse model under the optimal centrifugation parameters

**Fig. 7:**
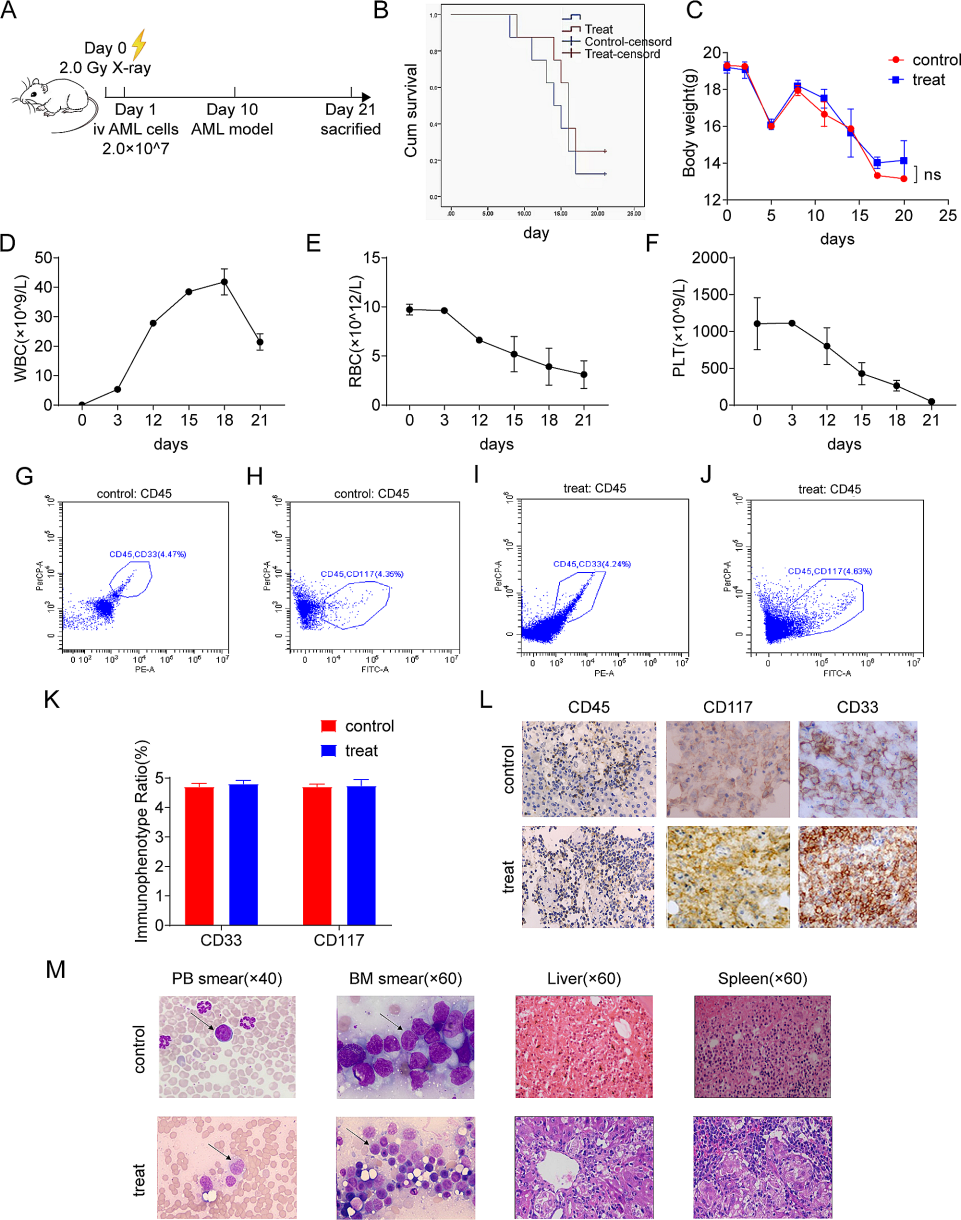
(**A**) A flow diagram of the leukemia mouse model (**B**) Survival curves of mice in the control and experimental groups (**C**) Changes in body weight of mice in the control and experimental groups (**D**) Changes in the number of white blood cells over time (**E**) Changes in the number of red blood cells over time (**F**) Changes in the number of platelets over time (**G - J**) The cell flow cytometry of immunophenotype ratio (**K**) Immunophenotype ratio of the control and experimental groups (**L**) Immunohistochemistry of the control and experimental groups (**M**) Staining smear of control and experimental groups

From the above results, we obtained the optimal centrifugation parameters (6000 rpm, 10 min) with minimal damage to cells. The leukemia cells centrifuged with the optimal centrifugation parameters were frozen, and their recovery activity reached more than 95% (Fig. 6C, D, E). Experimental cells were divided into experimental and control groups according to whether or not centrifugation was performed with the optimal parameters. Then they were injected into NSG mice by tail vein respectively, while the incidence cycle, infiltration and distribution of leukemia cells in mice were observed and compared. The results (Fig. 7) showed that there was no significant difference in weight changes and survival curves between the experimental group and the control group, and all mice successfully developed acute myeloid leukemia and died successively. CD45 positivity was seen by flow cytometry and immunohistochemistry, as well as the infiltration of leukemia cells could be seen in HE staining of the spleen and bone marrow. These results indicated successful establishment of leukemia mouse model and showed the aggressiveness of AML.

## Discussion

It is evident from our research that the most critical specific parameters in optimizing the leukapheresis process are the centrifugal speed and duration. If the centrifugal speed is too small or the duration is too short, the cell separation efficiency will be low. On the contrary, if the centrifugal speed is too high or the duration is too long, it will give the cells too much pressure and damage, and then cause cell rupture.

In the process of leukapheresis, on the one hand, the centrifugal speed may be too fast or the duration is too long, resulting in cell rupture, the release of a large number of harmful substances, and further damage to other cells. On the other hand, it can lead to a certain degree of platelet aggregation and activation. By optimizing leukapheresis and finding the optimal centrifugation parameters, the degree of cell rupture, platelet aggregation and activation can be reduced to a certain extent, so as to avoid aggravating the disease of patients.

For different components of blood cells, in order to separate them better, the parameter that is actually controllable and changed is the centrifugal speed and duration in the specific separation operation. After optimization, leukapheresis can promote the release of marginal leukemia cells into the blood vessels, thereby increasing the sensitivity of chemotherapy [21, 22]. On the other hand, it is possible to block the activity of certain myeloid leukemia cells during the synthetic phase, thereby enhancing the sensitivity of cell-specific drugs such as cytarabine or other synthetic phase specific drugs [23]. Our study suggests a high possibility that optimized leukapheresis can significantly and more rapidly reduce white blood cells, resulting in longer overall survival in patients with hyperleukocytic acute myeloid leukemia. After optimization, patients undergoing leukapheresis are more likely to have less cell damage, which means better long-term recovery and fewer complications.

A study found that centrifugal speeds had the greatest effect on the separation efficiency of the platelet separation efficiency model, while the effect on the separation efficiency of red and white blood cells at high centrifugal speeds was negligible [24]. Our study found that red blood cell morphology changed at 9000 rpm and above, while the loss of red blood cells at 6000 rpm was almost zero. But the range of changes in hemoglobin, the number of red blood cells and hematocrit had no statistical difference, which was consistent with literature reports.

However, platelets have different degrees of damage at high centrifugal speeds, so the optimal centrifugation parameters should also refer to the damage of platelets. Our study found that the number of platelets did not decrease significantly below 6000 rpm, while that decreased at 7500 and 9000 rpm. Platelet activation rate was within the normal range at 6000 rpm, and beyond the normal range at 7500 rpm and above. In addition, at 3000 and 6000 rpm, platelets had normal morphology under electron microscope with no foot process, fewer Alpha particles in cells, fewer mitochondrial vacuolation. The morphology of foot process elongated at 7500 and 9000 rpm. And at 12,000 rpm, there were more platelet Alpha particles and the platelet aggregation.

When centrifuged at 12,000 rpm, the biggest changes in platelet morphology were Alpha particles, dense particles and foot process. It has been documented that Alpha particles contain membrane binding proteins and soluble proteins that are involved in various processes, including cell adhesion, coagulation, inflammation, cell growth, and host defense [25]. After platelet activation, Alpha particles release fibrinogen and VWF, promoting platelet-platelet and platelet-endothelial cell interactions. In addition, components such as the fibrinogen receptor Alpha IIBβ3 found in Alpha particles are expressed on the surface of platelets and subsequently support platelet adhesion [25].

In this study, we tried for the first time to extract peripheral blood mononuclear cells with the optimal centrifugation parameters explored, and successfully froze cells with autologous plasma. The survival rate of cells after recovery was above 95%, which laid a solid foundation for the establishment of blood cell sample bank in the future.

We also successfully established the AML mouse model with the optimal centrifugation parameters. We know that animal models are indispensable research tools. For example, extensive sequencing efforts have mapped the genomes of adults and children with acute myeloid leukemia, revealing many biological and prognostic drivers. In addition to identifying recurrent gene aberrations, it is critical to adequately describe the complex mechanisms by which they contribute to the onset and evolution of disease, ultimately facilitating the development of targeted therapies. To achieve these goals, rapid advances in genetic engineering techniques over the past 20 years have greatly facilitated the use of mouse models to reflect specific genetic subtypes of human acute myeloid leukemia, define intracellular and external disease mechanisms, study interactions between co-occurring genetic lesions, and find new treatments. Therefore, it is of great significance to establish the AML mouse model. In this study we established the AML mouse model successfully using the cells obtained at the optimal centrifugation parameters. Not only these cells had less cell damage and improved the purity of mononuclear cells collected, but also the survival rate after cell cryopreservation and recovery was similar to that obtained by low-speed centrifugation. These results indicated that the centrifuged cells under the optimal centrifugation parameters could be used for cryopreservation of clinical samples and establishment of AML mouse models in laboratory.

However, our study still has certain limitations. For instance, we selected patients with hyperleukocytic acute myeloid leukemia who were all female and older, and different genders or ages may have some influence on the results. In addition, geographical distribution may also affect the results of the study, as environmental factors and lifestyle factors in different regions have a certain impact on the disease. Except for different patient demographics, our study is an invitro study analyzing centrifugation speeds and it can only hypothesise or comment on its possible adaptations to clinical leukapheresis. In order to make it constitute evidence, different centrifugation speeds need to be tested in vivo with different centrifugation speeds on leukapheresis.

## Conclusion

At present, the leukapheresis in clinical practice can not achieve the ideal effect, because it takes a long time and causes great damage to cells. It is an urgent problem to further improve leukapheresis, explore the optimal centrifugation parameters, realize fast and accurate white blood cell separation, and minimize the possible adverse reactions in the process. In this study, by changing the centrifugal speeds, it was observed that low-speed centrifugation was relatively safe for peripheral blood cells, while high-speed centrifugation would cause platelet activation and aggregation when it reached 12,000 rpm, and the possible optimal centrifugation parameters were found at 6000 rpm, 10 min. The cells obtained by centrifugation with the optimal parameters we explored could be frozen, and the survival rate was above 95%, and the AML mouse model could be successfully established.

The optimal centrifugation parameters found in this study can effectively reduce the number of white blood cells, while reduce the damage to red blood cells and platelets as much as possible, and ensure the cell survival rate. Therefore, what we have found has great potential to improve the efficiency of white blood cell apheresis therapy in clinical practice, and reduce the occurrence of related risks and complications.

The results of this study are of great significance for the improvement of clinical leukapheresis therapy, which is high likely improve the efficiency, reduce the treatment cost, guide the clinical prevention and treatment of adverse reactions in the process of leukapheresis therapy without increasing the risk related to treatment, and provide more scientific information for the evaluation of the collection effect of leukapheresis therapy and more scientific direction for the improvement of treatment instruments.

But it is worth mentioning that although leukapheresis is still the main means of treatment for hyperleukocytic acute myeloid leukemia, it is still hoped that there are better methods to replace or enhance the clinical practice, which needs to be further explored.

### Electronic supplementary material

Below is the link to the electronic supplementary material.

Supplementary Material 1

## Data Availability

The datasets used and/or analyzed during the current study are available from the corresponding author upon reasonable request.
