# Correction: Study on leukapheresis of hyperleukocytic acute myeloid leukemia through in vitro centrifugation

**DOI:** 10.1186/s12885-024-12702-y

**Published:** 2024-07-30

**Authors:** Ruiyang Pan, Anjie Xu, Li Liu, Jinxian Wu, Xinqi Li, Guopeng Chen, Ruihang Li, Wanyue Yin, Dandan Liu, Xiaoyan Liu, Fuling Zhou

**Affiliations:** Department of Hematology, Zhongnan Hospital of Wuhan University, 169 Donghu Road, Wuhan, 430071 P. R. China

**Correction: BMC Cancer 24, 888 (2024)**

10.1186/s12885-024-12644-5

Following publication of the original article [1], a typesetting error was reported, whereby Xiaoyan Liu was mistakenly not marked as a co-corresponding author. This has been corrected in this correction article.

The original article [1] has been corrected.
