# Supplementary material for: Study on leukapheresis of hyperleukocytic acute myeloid leukemia through in vitro centrifugation
Source: BMC Cancer. 2024 Jul 24;24:888. doi: 10.1186/s12885-024-12644-5 (PMC11267852; doi:10.1186/s12885-024-12644-5)
Supplement: Supplementary file 1 — Supplementary Material 1 [file 12885_2024_12644_MOESM1_ESM.pdf]

The information on the AML patients whose samples were used.

serial number	gender	WBC counts($\times 10^9$/L)	type of AML	amount of sample drawn
1	female	162.01	AML-M5	2ml per tube 10 tubes per person
2		107.05	AML-M4	
3		263.93	AML-M1	
4		393.2	AML-M1	
5		226	AML-M2	
6		224.4	AML-M1	
7		189.61	AML-M5	
8		178.7	AML-M1	
9		151.5	AML-M5	
10		116.3	AML-M1	
11		106.67	AML-M5	
